# Molecular Mechanisms of Accelerated Ageing in Geriatric Depression: Interplay of Telomere Attrition, Mitochondrial Dysfunction and Cellular Senescence

**DOI:** 10.3390/ijms27031613

**Published:** 2026-02-06

**Authors:** Pratibha Revi Shanker, Rajkumar Dorajoo

**Affiliations:** 1 Genome Institute of Singapore (GIS), Agency for Science, Technology and Research (A*STAR), 60 Biopolis Street, Genome, Singapore 138672, Singapore; 2 Department of Paediatrics, Yong Loo Lin School of Medicine, National University of Singapore, Singapore 119228, Singapore

**Keywords:** depression, telomere length, mitochondria, apoptosis, inflammation

## Abstract

Late-life depression is a prevalent and debilitating disorder. It differs significantly from depression in younger adults and often co-occurs with cognitive decline and increased physical frailty. This narrative review explores the role of accelerated biological ageing in late-life depression. We examine evidence linking three interconnected processes, namely telomere attrition, mitochondrial dysfunction and cellular senescence, to the pathophysiology of late-life depression. Excessive attrition of telomeres may serve as a biomarker of accumulated stress and cellular ageing. Mitochondrial dysfunction not only reduces energy production but also promotes oxidative stress and inflammation that increase neuroinflammatory pathways and synaptic loss. Increased cellular senescence further induces senescence-associated secretory phenotype factors that drive chronic inflammation and neuronal loss. Together, these processes create a cycle of cellular stress, persistent inflammation and damage to brain circuits involved in late-life depression. We additionally highlight potential limitations in current findings and propose a roadmap for future research to better elucidate the mechanistic dysfunction of late-life depression. These include the need for evaluation in long-term prospective cohort studies, improved tools to better correlate blood-based markers with changes in disease-relevant brain tissues and regions, and trials that test treatment and lifestyle modifications that are targeted at ageing biomarkers.

## 1. Introduction

Increasing age is accompanied by a gradual deterioration of multiple biological functions, and this time-linked loss of physiological resilience remains the most powerful predictor of major chronic illnesses such as cardiovascular disease, cancer and neurodegenerative conditions [1]. To explain why ageing predisposes individuals to such a broad spectrum of diseases, the hallmarks of an ageing framework offer a cohesive model that brings together core mechanisms driving cellular decline. These hallmarks include genomic instability, progressive telomere shortening and mitochondrial impairment, and have recently been expanded to incorporate chronic, low-grade inflammation and disruptions in gut microbial composition [2,3]. Rather than acting independently, these processes interact across molecular, cellular and systemic levels. DNA damage disrupts gene regulation, mitochondrial inefficiency increases oxidative stress, and immune dysregulation fuels persistent inflammation. Over time, these interconnected biological disturbances weaken the capacity to maintain homeostasis and capability to effectively respond to physiological challenges, creating a landscape of heightened vulnerability to disease in later life.

Late-life depression (LLD) is an increasing public health concern with significant implications for ageing populations worldwide. It is one of the most common psychiatric conditions affecting older adults aged 65 years and above worldwide. The global prevalence of LLD is estimated to be approximately 13%. However, rates of clinically relevant depressive symptoms exceed 40% in people living in long-term care settings or in those with significant comorbidities [4,5]. Prevalence is shaped by several factors such as living conditions, medical status, social connections, and gender differences. Affected individuals experience greater functional decline, poorer treatment response, and increased risk of cardiovascular events, cognitive deterioration and all-cause mortality compared with younger adults with depression. Compared with depression that begins earlier in life, LLD is more strongly influenced by the burden of chronic medical conditions, use of multiple medications and reduced social engagement, while depression in younger adults is more strongly linked to genetics, early life stress and trauma [6,7]. Distinguishing between early-onset depression that recurs in later life and depression that first appears in older age is clinically important, as late-onset cases are more strongly linked to comorbidity and neurodegenerative processes [8].

A growing body of research suggests that ageing biology and LLD involve overlapping biological dysfunctions [9,10,11]. Mitochondrial ROS-induced cellular damage may accelerate telomere shortening and senescent cell accumulation. Senescent cells release pro-inflammatory senescence-associated secretory phenotype (SASP) factors, driving neuroinflammation and disruption of brain circuits involved in mood regulation.

Table 1 provides an overview of findings from both human studies and in vivo animal models that support this accelerated-ageing perspective in LLD. Understanding how these ageing mechanisms contribute to depressive illness is timely and necessary. Age-related mitochondrial dysfunction, telomere maintenance and immune regulation directly affect the integrity of neural circuits involved in emotion regulation and cognition. These processes contribute to heightened susceptibility to both neurodegenerative and mood disorders, highlighting the need to conceptualise LLD not simply as a psychiatric condition, but also as a disorder that may be rooted in broader systemic ageing mechanisms. As many nations face rapid demographic transitions toward an older population, clarifying these pathways will help improve diagnostic accuracy, support early identification of those at elevated risk and guide the development of intervention strategies that target the underlying biology rather than only the symptoms of depression [12].

**Figure 1:**
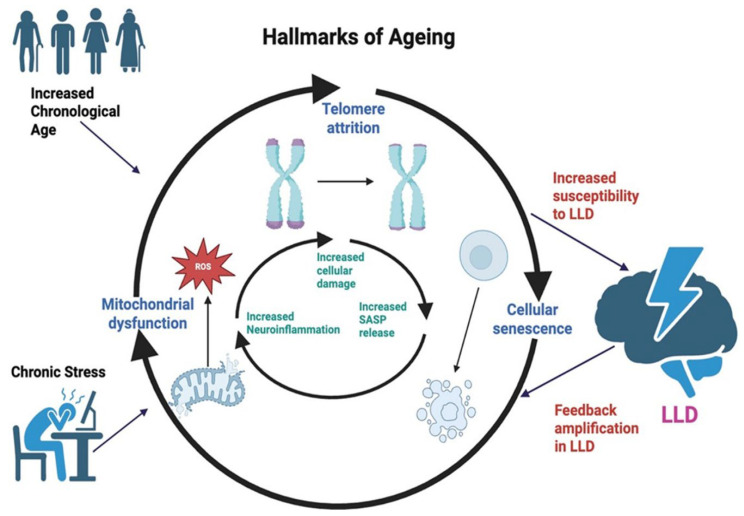
Interacting hallmarks of ageing in late-life depression (LLD). Increased chronological age and chronic stress promote mitochondrial dysfunction, telomere attrition, and cellular senescence, key hallmarks that increase vulnerability to LLD. The schematic illustrates the integrated and mutually reinforcing nature of ageing-related mechanisms in LLD rather than isolated pathways. Mitochondrial dysfunction and ROS-induced damage accelerate telomere shortening and senescent cell accumulation. Senescent cells secrete SASP factors, driving neuroinflammation and impairing neural circuits involved in mood regulation. In turn, LLD-associated stress and inflammation may further exacerbate mitochondrial dysfunction and senescence in brain regions, creating a self-reinforcing feedback loop that amplifies biological ageing and depressive pathology.

In this review, we focus on three major and interlinked hallmarks of ageing, namely telomere attrition, mitochondrial dysfunction and cellular senescence, that increase susceptibility to LLD (Figure 1). Together, these processes promote a self-reinforcing cycle of physiological stress, immune imbalance and disruption of brain circuits involved in mood regulation. Despite growing recognition of these biological links, the underlying molecular pathways that connect ageing processes to depressive symptoms remain poorly defined. Existing studies often treat ageing hallmarks as isolated processes rather than an integrated network. This review brings together evidence from human and animal studies to clarify how these intertwined mechanisms contribute to LLD, and to identify opportunities for prevention and therapy grounded in geroscience principles.

## 2. Telomere Attrition: An Indicator of Long-Term Stress and Increased LLD Susceptibility

Telomere attrition refers to the progressive loss of DNA sequences at the ends of chromosomes during cell division. This is due to the inability of DNA polymerase to fully replicate chromosome ends, also known as the end-replication problem. Attrition of telomeres is further accelerated due to damage from oxidative stress and inflammation [13]. As telomeres progressively shorten, they lose their protective function, leading to DNA damage response, cellular senescence or apoptosis. This eventually restricts the regenerative capacity of tissue-specific stem cells. Post-mitotic cells, such as neurons, can also enter a senescence-like state triggered by persistent telomeric DNA damage rather than traditional telomere shortening [14]. Telomere length is especially responsive to psychosocial burden and persistent oxidative injury. Frequent exposure to psychological and physiological stressors is common in older adults who experience depressive symptoms [15,16,17]. As the length of telomeres in circulating immune cells (i.e., leukocytes) gradually declines in response to long-term stress, telomere length measured from leukocytes (LTL) may also serve as a biological record of adversity accumulated across decades of life [15,16,17].

While LTL correlates with age-related morbidity and mortality, its relationship to region-specific neuronal ageing in LLD has not been firmly established, and direct evidence demonstrating telomere attrition within specific brain regions implicated in LLD, such as the hippocampus and prefrontal cortex, remains limited. However, circulating whole blood cell TL has been shown to be correlated with TL in other tissues, including brain regions such as the hippocampus [18,19]. Additionally, LTL can be an indicator of systemic oxidative stress, which may also impact neuronal cells and brain-related processes [20].

Recent research has explored the link between LTL attrition and geriatric depression (Table 1). Findings showed that older adults reporting mild depressive features or subtle cognitive concerns already exhibited reduced telomeres in their blood leukocytes [21]. In the study, shorter LTL was also observed to be associated with increased cytokine levels (such as interleukin-6 levels) [21]. It may be possible that increased immune activity and corresponding increases in inflammation levels contribute to greater attrition of telomere reserves over time, leading to an immunoaging phenotype. Rather than simply being a biomarker for LLD, it may be likely that increased telomere attrition could reveal an underlying biological fragility that precedes the onset of disease. Data from a prospective cohort study corroborates this. Prolonged exposure to psychosocial stress was shown to be associated with increased telomere attrition over time as compared to individuals with more acute fluctuations in an emotional state [22].

While associations between LLD and telomere length levels have been reported, whether telomere attrition leads to or is a causative factor for the onset of LLD remains uncertain. Current findings nevertheless support the view that shorter telomeres signal a pre-existing, accelerated ageing state that increases the likelihood of developing LLD along with cognitive and physical problems that commonly accompany the condition [21,22]. Telomere-related DNA damage response may steer cells towards senescence and amplify inflammatory signaling through SASP factors [23,24]. These inflammatory mediators may impair vascular and neural function, processes considered central to LLD pathophysiology [25,26]. Increased oxidative stress generated by impaired mitochondria may also intensify telomere shortening, linking this ageing biomarker with mitochondrial dysfunctions [27,28], which is discussed in the following section. Taken together, telomere attrition appears to function primarily as a biological record of accumulated stress and cellular wear and tear. As such, it may help identify older adults who are at elevated risk for LLD and other related disorders that emerge during the ageing process.

## 3. Mitochondrial Dysfunction: Declining Energy Production and Escalating Inflammatory Signaling

Ageing brings gradual failure in many aspects of mitochondrial activity. Cells begin to show weaker oxidative metabolism, compromised mitophagy and increased formation of reactive oxygen species (ROS) [29]. These changes limit the supply of energy (ATP) in the cells and increase cellular stress, which in turn increases the risk of tissue damage and neurodegenerative processes [30,31]. Within the ageing nervous system, these mitochondrial dysfunctions affect synaptic plasticity, lower the capacity of neurons to withstand stress and reduce the function of glial cells [32,33,34]. All these functions contribute directly to mood stability and emotional control. As mitochondria respond to hormonal stress cues and changes in cellular redox balance, they serve as key regulators of how the body processes psychological and biological stressors [35,36,37,38].

Impairments in the mitochondria can promote depressive symptoms through several routes. Neurons receive less energy to maintain their structure and signaling, and damage due to oxidative stress accumulates in proteins, lipids, nucleic acids and other cellular components. Stress-related and inflammatory pathways also become more active, creating a biological environment that favours depressive states in LLD [39,40]. Recent studies on older adults offer support for this association [41,42,43] (Table 1). Individuals with LLD are observed with an increased burden of mitochondrial DNA (mtDNA) damage within circulating exosomes and are also associated with both declining cognitive performance and higher levels of inflammatory markers [41]. It is suggested that this damaged mtDNA that is released into circulation may act as a damage-associated molecular pattern (DAMP), amplifying neuroinflammatory signaling. Additional studies in adults with both LLD and frailty also show increased levels of circulating cell-free mtDNA. This indicates more severe cellular stress and mitochondrial damage that may have been synergistically amplified due to the co-existence of both the frailty and depression conditions in these individuals [42]. Furthermore, molecular evidence from people with major depressive disorder indicates disruption of how mitochondria divide and merge. Interruption of the balance of mitochondrial repair, mitophagy and replacement results in the accumulation of dysfunctional mitochondria that release proinflammatory signals that may damage neurons [43]. The biological disturbances linked to LLD reflect more than a simple reduction in energy production and may indicate a broader collapse in the systems that normally protect mitochondrial stability and overall cellular health.

Mitochondrial dysfunctions also initiate and interact with other ageing-related processes. When cells that shift into a senescent state hold on to damaged mitochondria instead of clearing them effectively, excessive reactive oxygen molecules are produced. These mitochondria intensify the senescence programme and increase the release of inflammatory SASP factors [44]. At the same time, oxidative stress from dysfunctional mitochondria accelerates attrition of telomeres. This interplay creates a cycle in which damage to mitochondria and erosion of telomeres reinforce one another [45,46]. As a result, mitochondrial dysfunction becomes a driver of widespread inflammageing, shaping immune exhaustion, vascular instability and altering brain–immune communications [47,48]. A further consequence is the release of mtDNA into the extracellular space. This has been shown to bind to receptors such as factors such as Toll-like receptors (for e.g., TLR9) on microglia and astrocytes, activating neuroinflammatory pathways in the brain and weakening neuroplasticity and networks that support mood regulation [41,42,49].

Interventions that stabilise mitochondrial function and raise cellular NAD (Nicotinamide Adenine Dinucleotide) levels are considered possible options for improving mood and cognition in older adults [50,51]. These studies are supported by several preclinical studies that highlight improvements to depression phenotypes (i.e., sucrose preference and forced swimming tests) in animal models [52,53,54,55]. Such interventions that are targeted at mitochondria or mitochondrial components are aimed at not only reducing depressive symptoms but also addressing the underlying physiological decline that often accompanies LLD. Preliminary findings suggest that these approaches are generally well tolerated in older adults and may improve bioenergetic efficiency and inflammatory profiles; however, evidence for direct antidepressant efficacy in LLD remains inconclusive [56,57,58].

## 4. Cellular Senescence: A Unifying Engine of Systemic Ageing

Cellular senescence refers to a stable and irreversible arrest of the cell cycle that occurs when cells experience substantial stress or damage. Triggers include critically shortened telomeres, persistent DNA damage, oxidative injury and the inappropriate activation of oncogenic pathways. Although this response is protective in the short term and prevents damaged cells from proliferating and contributing to tumour formation, the progressive build-up of senescent cells with advancing age becomes harmful [59,60]. Rather than remaining inert, senescent cells have been characterised as being metabolically active and undergo extensive changes in their secretory profile. They release a broad array of inflammatory cytokines, chemokines, growth modulators and matrix-remodeling enzymes collectively referred to as the senescence-associated secretory phenotype (SASP) [59,60]. This secretome has wide-ranging effects. It promotes chronic low-grade inflammation, disrupts tissue architecture, alters the extracellular matrix integrity and affects stem cell renewal [61,62]. Through paracrine signaling, SASP factors also propagate senescence to surrounding cells, resulting in an expansion of the senescent cell burden within tissues [63]. Over time, this creates an increasingly inflammatory and dysfunctional microenvironment that undermines tissue and organ health. In the brain, SASP-mediated inflammation can impair neurogenesis, reduce synaptic plasticity and heighten the activation of microglia and astrocytes [63,64,65,66]. These processes may collectively erode resilience in neural circuits associated with mood regulation. These cumulative effects make cellular senescence a central biological contributor to the vulnerability observed in late-life mental health conditions, including depression.

Chronic stress, a key risk factor for depression, accelerates cellular senescence in the brain. Animal model studies have demonstrated that increased stress induces senescence markers, such as p16INK4a, in the hippocampus [67,68,69,70,71,72] (Table 1). These senescent cells remain metabolically active and accumulate with stress exposure, leading to depression-like behaviour. Pro-inflammatory senescence-associated SASP was identified as a primary mechanism, resulting in chronic inflammation, impairing tissue repair and disrupting neural circuits critical for mood regulation.

Importantly, senescent cells can be selectively eliminated using genetic ablation systems such as p16-INK4a-driven suicide gene in B6 mice (INK-ATTAC) [68,69,70,71,72]. Such an approach enables temporal control of senescent cell clearance and allows direct testing of causality between senescence and behavioural outcomes. Clearance of the senescent cells from the experimental models produced reversal of phenotypic effects and improvements in cognitive functions, such as nest building (an indicator of hippocampal-integrated executive cognitive function) and improvements to the water-motivated stone maze task that probes hippocampal-related decision-making, learning, and memory [72]. This highlights that targeting these senescent cells represents a mechanistically grounded and experimentally validated strategy for stress-related mood disorders [73].

A recent human study also suggested that accumulation of cellular senescence is a key biomarker in age-related depression [74] (Table 1). Cell senescence levels were higher among individuals with depression [74]. The study further suggested that targeting senescence may be particularly effective for individuals with dual comorbidities of cognitive and physical decline, which are known to worsen the prognosis for adults with LLD [74]. The clinical manifestation of this process is the “depressed frail phenotype,” which is the simultaneous occurrence of LLD and physical frailty [75]. Co-occurrence of frailty and depression leads to increased risks of earlier mortality and worsens cognitive decline [76]. Thus, identifying individuals who are predisposed to physical frailty may serve to subtype LLD, identifying a subset of high-risk individuals who may require closer monitoring and more aggressive treatment strategies.

From the therapeutic angle, there is rising interest in senolytics that target senescent cells and senomorphics that regulate their secretory products. Preclinical studies have demonstrated that these approaches can reduce senescent cell burden and downstream inflammation, most notably using dasatinib–quercetin combinations in ageing and stress-related animal models [77]. Early-phase human studies have also begun to explore the translational potential of senolytic therapies, with small-scale pilot trials indicating that such interventions are tolerable in older adults and may be associated with reductions in circulating SASP factors and pro-inflammatory cytokines (such as TNF-alpha), as well as improved physical and cognitive performance [78,79]. However, these findings remain preliminary and larger and better-powered clinical trials with neuropsychiatric endpoints will be required to establish efficacy, optimal dosing, and long-term safety in LLD.

## 5. Limitations and Future Directions

Despite compelling evidence linking ageing biology to LLD, several limitations restrict the field’s ability to draw causal inferences or translate findings into clinical practice (Table 2). Recent longitudinal data suggest a potential bidirectional relationship between biological age acceleration and depressive symptoms [80]. A major constraint is the predominance of cross-sectional study designs, which makes it difficult to establish the temporal sequence of events, specifically whether markers of accelerated ageing precede and contribute to the onset of LLD or emerge as a consequence of the disorder. Additional longitudinal studies that repeatedly measure ageing-related biomarkers, such as telomere length, SASP components and indices of mitochondrial performance, in initially non-depressed older adults would help identify which markers predict future depressive episodes and clarify whether biological ageing precedes mood deterioration.

In parallel, analytical methods capable of probing causality represent an important next step. Mendelian Randomisation (MR) leverages germline genetic variants as instruments for lifelong exposure to biological age acceleration effects [81,82,83] and provides a framework to test whether inherited genetic predisposition to shorter telomere length, altered mitochondrial function or heightened inflammatory activity contributes to LLD risk. Findings from these approaches could complement observational evidence and reduce effects of confounding factors, strengthening causal inferences. To date, such genetically informed analyses remain limited for LLD [81]. Recent growth in large-scale genome-wide association studies has markedly expanded the availability of genetic variants for ageing-related traits [84,85,86], offering new opportunities to improve MR analyses and integrate ageing biology with LLD risks.

A second major challenge is that evidence supporting the relevance of circulating age-related biomarkers to central neuroinflammatory and neurodegenerative processes remains limited [87]. There is a need for direct evaluation of biological ageing processes in the disease-relevant central nervous system tissues. Approaches that combine imaging methods, cerebrospinal fluid assessments and analysis of brain tissue together with matched blood samples could help determine the reliability of circulating biomarkers to reflect neuronal ageing processes. Additionally, newer strategies, such as isolating neuron-derived extracellular vesicles, provide a strategy to sample molecular signals originating specifically from neurons and may improve understanding of the mechanisms of ageing-related brain changes [88,89,90].

Interpretation of current findings is further complicated by the considerable heterogeneity that characterises LLD. Older adults with depressive symptoms do not represent a single uniform biological or clinical group. Some individuals show features consistent with vascular-driven depression, where cerebrovascular disease and disrupted white-matter integrity impair fronto-subcortical circuits [91]. Others display an inflammation-related presentation, marked by elevated cytokines and metabolic dysregulation. A separate group demonstrates more classical melancholic symptoms, such as psychomotor changes and profound anhedonia, while another subset expresses depressive symptoms that may serve as early indicators of evolving neurodegenerative processes [10]. The varied phenotypic expression suggests that LLD is not a homogeneous disorder but encompasses multiple biological subtypes that converge on a shared clinical endpoint.

To address this complexity, more sophisticated approaches to patient stratification are needed. Development of composite biological ageing scores that incorporate telomere dynamics, mitochondrial health, and senescence markers, as well as other important biomarkers of ageing such as epigenetic clocks [92,93], may enable more precise stratification strategies. Combining these molecular datasets with neuroimaging, cognitive measures, vascular markers, and psychosocial context may lead to a more accurate classification of LLD that reflects distinct disease mechanisms rather than relying solely on symptom-based criteria. Ultimately, biologically informed stratification may pave the way for targeted interventions and personalised treatment strategies that address the underlying ageing pathways unique to each subgroup.

Epidemiological evidence highlights the influence of modifiable lifestyle behaviour on the risk of LLD. Regular physical activity, higher consumption of omega-3 fatty acids and adherence to nutrient-dense dietary patterns consistently correlate with lower rates of depressive symptoms in older adults [94,95,96]. Importantly, this behaviour is not only protective at the psychological or behavioural level, but may also exert measurable effects on fundamental ageing pathways [97,98,99]. Exercise improves mitochondrial turnover through enhanced mitophagy, reduces systemic inflammation and supports neuroplasticity [100]. Similarly, omega-3 fatty acids have been shown to modulate membrane composition, decrease oxidative stress, and attenuate production of pro-inflammatory cytokines [101,102]. Epidemiological studies also indicate that populations with diets rich in antioxidant-containing foods, such as the Mediterranean diet, exhibit a lower prevalence of depressive symptoms in older age [103,104,105].

Studies that combine structured lifestyle modifications with prospective and repeated assessments of key biological ageing indicators are required and may demonstrate that lifestyle-driven improvements in these ageing biomarkers directly mediate lower LLD incidence. Such evidence could transform lifestyle recommendations from general health advice into targeted, geroscience-informed interventions capable of modifying the biological trajectory that predisposes older adults to depression. This shift would support a prevention-oriented framework in which behavioural strategies are deployed, not only to enhance overall well-being but also to directly counteract age-related molecular processes that undermine mood and cognitive function.

Coordinated efforts are required to bring together fundamental biological research, prospective cohort study evaluation and intervention trials. Integration of biomarkers of ageing hallmarks with improved neuroimaging, genomics and digital phenotyping allows researchers to build a more precise biological model of LLD. This integrated framework has the potential to reveal how age-related cellular and molecular processes shape the onset and trajectories of depressive symptoms. A clearer understanding of these causal pathways would ultimately support a transition away from approaches that focus solely on symptomatic management and towards strategies that emphasise prevention, early detection, and personalised treatment based on an individual’s biological ageing profile.

## 6. Conclusions

This review highlights that LLD is closely intertwined with fundamental processes that drive biological ageing. Attrition of telomeres, increased mitochondrial dysfunction and the accumulation of senescent cells do not simply reflect the passage of time; they actively shape the physiological environment that can give rise to depressive symptoms. Together, these processes generate a self-perpetuating loop in which long-term stress and molecular injury promote energy imbalance, persistent immune activation and disruption of neural systems that regulate mood and cognition. Additional research is required to clarify causal mechanisms, identify circulating and brain-specific markers that enable the reliable tracking of disease processes, and biologically categorise the heterogeneous presentations of LLD. At the same time, rigorous evaluation of treatments that target ageing-related pathways is required. Approaches aimed at reducing senescent cell burden, enhancing mitochondrial resilience or improving cellular stress responses represent promising strategies to mitigate the biological dysfunctions that drive vulnerability in older adults.

There is an urgent priority to integrate geroscience principles into mental health research and clinical practice, especially with an increasing proportion of ageing populations worldwide. Addressing these upstream biological dysfunctions that contribute to LLD represents potential not only to reduce depressive symptoms but also to meaningfully extend functional capacity to improve quality of life as population ageing increases globally.

## Figures and Tables

**Table 1:** Human and experimental evidence supporting the role of telomere attrition, mitochondrial dysfunction and cellular senescence in late life depression.

Hallmark	Molecular Alterations (Features)	Neuronal Consequences
Telomere Attrition	- Shortened leukocyte telomere length - Increased telomere-associated DNA damage response (DDR)	Activation of p53-mediated senescence pathways resulting in reduced cellular viability and regenerative capacity. Triggers the cellular senescence program and increases vulnerability to age-related cognitive and mood disturbances.
Mitochondrial Dysfunction	- Elevated circulating cell-free mtDNA (ccf-mtDNA) - Increased mitochondrial DNA deletions detected within circulating exosomes - Elevated markers of oxidative stress (e.g., ROS)	Bioenergetic failure in neurons and glial cells releases excess reactive oxygen species (ROS) Mitochondrial DNA (mtDNA) leakage, which activates neuroinflammatory pathways via DAMP signaling. (e.g., TLR9) Heightened neuronal apoptosis and synaptic loss, which worsens bioenergetic failure.
Cellular Senescence & SASP	- Upregulated senescence markers (p16INK4a, SA-β-gal) - Elevated levels of systemic and CNS-localised SASP factors (IL-6, IL-1β, TNF-α)	SASP-driven microglial and astrocyte activation that sustains chronicneuroinflammation and disruption of neural circuitry. Impaired hippocampal neurogenesis and synaptic plasticity. The promotion of white matter deterioration through inflammatory and oxidative pathways.

**Table 2:** Roadmap to address major current limitations in the field of biological ageing and LLD.

Current Challenge	Proposed Study Design/Methodology	Translational Goal
Establishing Causality	Cohort studies: Longitudinal cohorts that track age-related biomarkers in non-depressed older adults. Genetic studies: Mendelian randomization studies using genetic risk variants (from GWAS studies) associated with age-related biomarkers such as telomere length attrition and mitochondrial levels.	Prove that temporal and causal pathways between ageing hallmarks precede and cause LLD.
Bridging the Peripheral-Brain Gap	Imaging-biomarker integration: Correlate blood markers with translocator protein PET neuroimaging for neuroinflammation and neurodegeneration measures. Multi-level tissue analyses: Parallel quantification of biomarkers in matched blood, CSF, and post-mortem brain tissue. Neuron-derived proxies: Develop and validate neuronally derived extracellular vesicles (NDEVs) as blood-based brain proxy indicators of neuronal ageing.	Validate that circulating biomarkers reflect CNS brain ageing. Develop reliable, non-invasive diagnostics for CNS brain pathology.
Deconstructing Heterogeneity	Data-driven cluster biological stratification: (e.g., latent class analysis) of large LLD cohorts. Symptom-biology mapping: Link specific biomarkers to symptom domains. (e.g., anhedonia, fatigue, cognitive slowing) Frailty-ageing interactions: Empirically validate the “depressed frail phenotype” against multi-modal biomarker profiles such as molecular, clinical and imaging signatures	Define and identify biotypes (e.g., “inflammatory-senescent” subtype). Enable precision psychiatry by matching patients to tailor specific therapies.
Translating to Treatment	Biomarker-guided therapeutics: Use baseline ageing biomarkers to predict response to conventional antidepressants, integrate biological ageing measures using longitudinal intervention studies for modifiable lifestyle changes.	Develop novel, biologically targeted therapies for ageing mechanisms. Personalize treatment selection to improve outcomes and remission rates of LLD.

## Data Availability

No new data were created or analyzed in this study.
